# Annexin A3 in sepsis: novel perspectives from an exploration of public transcriptome data

**DOI:** 10.1111/imm.13239

**Published:** 2020-08-31

**Authors:** Mohammed Toufiq, Jessica Roelands, Mohamed Alfaki, Basirudeen Syed Ahamed Kabeer, Marwa Saadaoui, Arun Prasath Lakshmanan, Dhinoth Kumar Bangarusamy, Selvasankar Murugesan, Davide Bedognetti, Wouter Hendrickx, Souhaila Al Khodor, Annalisa Terranegra, Darawan Rinchai, Damien Chaussabel, Mathieu Garand

**Affiliations:** ^1^ Sidra Medicine Doha Qatar

**Keywords:** annexin, bacteremia, cell proliferation, endotoxemia, immunity, inflammation, neutrophil, sepsis, transcriptome

## Abstract

According to publicly available transcriptome datasets, the abundance of Annexin A3 (ANXA3) is robustly increased during the course of sepsis; however, no studies have examined the biological significance or clinical relevance of ANXA3 in this pathology. Here we explored this interpretation gap and identified possible directions for future research. Based on reference transcriptome datasets, we found that ANXA3 expression is restricted to neutrophils, is upregulated *in vitro* after exposure to plasma obtained from septic patients, and is associated with adverse clinical outcomes. Secondly, an increase in ANXA3 transcript abundance was also observed *in vivo*, in the blood of septic patients in multiple independent studies. ANXA3 is known to mediate calcium‐dependent granules–phagosome fusion in support of microbicidal activity in neutrophils. More recent work has also shown that ANXA3 enhances proliferation and survival of tumour cells via a Caspase‐3‐dependent mechanism. And this same molecule is also known to play a critical role in regulation of apoptotic events in neutrophils. Thus, we posit that during sepsis ANXA3 might either play a beneficial role, by facilitating microbial clearance and resolution of the infection; or a detrimental role, by prolonging neutrophil survival, which is known to contribute to sepsis‐mediated organ damage.

## Graphical abstract

The expression of Annexin A3 (ANXA3), a member of a family of calcium‐binding proteins, is restricted to neutrophils. Its abundance is increased in septic patients, yet the functional relevance of ANXA3 in this context remains unknown. Upon reviewing evidence from public transcriptome repositories and the literature, we hypothesize that during sepsis ANXA3 could mediate neutrophils microbicidal activity while also prolonging their survival, and thus also possibly contributing to organ damage.

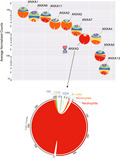

**Abbreviations**
ANXA3: Annexin A3
COD: collective omics data
GEO: Gene Expression Omnibus
PBMCs: peripheral mononuclear cells

## Introduction

This review article intends to explore a possible role for Annexin A3 (ANXA3) in the immunopathology of sepsis. Before attempting to do so by bringing forward original experimental evidence, the wealth of publicly available transcriptomic data can be leveraged to: (i) determine if changes in abundance of ANXA3 measured in earlier transcriptome profiling studies are robust; (ii) draw inferences regarding the potential biological significance or clinical relevance of such changes; and (iii) identify paths for future investigations.

A hands‐on workshop held at Sidra Medicine (Qatar) focused on the discovery of novel candidate genes implicated in the pathogenesis of sepsis. Public omics data were used as a source of training material for target selection and gene‐centric reductionist investigations *in silico*. The approach was described in a recent review (‘collective omics data’ training module 1 or COD1).^1^

Annexin A3 (ANXA3) was selected among a pool of candidates on the basis of: (i) its expression being increased *in vitro* in neutrophils exposed to plasma from a septic patient for 6 hr (GEO dataset GSE49755);^2^ and (ii) the apparent lack of literature addressing the role of ANXA3 in sepsis (the selection process is described in more details in Ref. [1]).

Annexins are calcium‐binding proteins: they bind negatively charged phospholipid membranes in a calcium‐dependent manner. These proteins are characterized by a conserved ‘core domain’ consisting of Ca^2+^‐binding motifs‐containing Annexin repeats. Annexins are involved in membrane transport and can mediate a wide range of cellular processes, including endocytosis, exocytosis and membrane‐cytoskeletal organization.^3^ As discussed below, ANXA3 promotes neutrophil granules aggregation in a calcium‐dependent manner, is involved in granule–granule and granule–phagosome fusion,^4^, ^5^ and also participates in the microglial response to motor nerve injury, blood vessel formation and adipocyte differentiation.^6^, ^7^, ^8^

## Materials and methods

### Reference datasets

Details about the study characteristics are thus provided here in addition to being summarized in Table 1. GSE49758 Khaenam *et al*.:^2^ A Transcriptomic Reporter Assay Employing Neutrophils to Measure Immunogenic Activity of Septic Patients Plasma. The datasets that were used as a starting point to select ANXA3 for further investigation were contributed by our group.^2^ Specifically, the GEO super‐series GSE49758 comprised three series (independent experiments): GSE49755, GSE49756, GSE49757. In this work, neutrophils, dendritic cells and peripheral blood mononuclear cells (PBMCs) were analysed by reporter assay to ‘sense’ the immunomodulatory factors present in the serum of septic plasma.^2^ Cells were isolated or derived from healthy donors, and cultivated *in vitro* in the presence of plasma from control and septic subjects. Diagnosis of sepsis was based on accepted international guidelines, and defined as presentation with two or more of the following criteria for the systemic inflammatory response syndrome: fever (temperature > 38° or < 36°), tachycardia (heart rate > 90 beats/min), leucocytosis or leucocytopenia (white blood cell count ≥ 12 × 109/l or ≤ 4 × 109/l). Blood was collected within 24 hr following the diagnosis of sepsis. Samples were selected from subjects who had the diagnosis of bacteremic sepsis retrospectively confirmed by the isolation of a causative organism on blood culture. Changes in transcript abundance were measured on a genome‐wide scale using Illumina Beadarrays. The findings were indeed consistent with the notion that neutrophils are less active transcriptionally, with lower repertoire diversity and lower RNA content (21 236 transcript species detected versus 25 728 in PBMCs and 23 589 in dendritic cells). Neutrophils proved nevertheless more responsive, with 823 transcripts differentially expressed versus 610 in PBMCs and 613 in dendritic cells. In addition, the extent of the transcriptional changes observed in neutrophils exposed to septic plasma correlated with the extent of the disease severity. The datasets were deposited and GEO and subsequently loaded by our team into a data‐browsing application (links are provided in Table 1, the GXB application has been described earlier in: Ref. [9]).

GSE30119 Banchereau *et al*.:^10^ Host immune transcriptional profiles reflect the variability in clinical disease manifestations in patients with Staphylococcus aureus infections. The goal of this study was to characterize changes in transcript abundance in the whole blood of paediatric patients with acute S. aureus infection. Previous work had instead used PBMCs — a cellular fraction that excludes neutrophils, which are essential for the immune response to bacterial infections. Blood was collected from hospitalized paediatric patients with culture‐confirmed *S. aureus* infection (*n* = 99) and from healthy controls (*n* = 44), at a single time point. Any subject with evidence of a viral infection was excluded. Enrollment took place at a single institution in the USA. The rather large cohort of subjects permitted an investigation into the differences in transcript abundance associated with the infection site, clinical presentation and/or disease severity. Samples were run on Illumina HT‐12 v3 beadarrays.

GSE64457 Demaret *et al*.:^11^ Marked alterations of neutrophil functions during sepsis‐induced immunosuppression. This study aimed to produce an in‐depth molecular characterization of neutrophils during the late immunosuppressed phase of sepsis. Enrollment took place at a single institution in France. Transcriptome profiles were generated from neutrophils isolated from nine adult septic shock patients at two time points (between 3 and 4 days, and between 6 and 8 days after sepsis onset) and from eight healthy volunteers. The patients presented with characteristics of injury‐associated immunosuppression, with reduced HLA‐DR expression, a low CD4+ lymphocyte count and an increased abundance of regulatory T‐cells.

GSE25504 Dickinson *et al*.:^12^ Whole blood mRNA expression profiling of host molecular networks in neonatal sepsis. The aim of this study was to use blood transcriptome profiling to better understand the immune response elicited by human neonates to infection. Samples were collected from subjects at the first suspicion of a clinical infection. Samples were retrospectively selected for analysis based on a positive blood culture. Enrollment took place at a single institution in the UK. We used the training set from this study to validate differences in ANXA3 abundance. This training set consisted of 27 patients and 35 age‐matched controls; the samples were run on Illumina HT‐12 V3 beadarrays.

GSE13015 Pankla *et al*.:^13^ Genomic Transcriptional Profiling Identifies a Blood Biomarker Signature for the Diagnosis of Septicemic Melioidosis. This study was also contributed by our group. We aimed to identify blood transcriptional signatures that differentiate septic patients infected by Burkholderia pseudomallei (the bacillus causing Melioidosis) from septic patients infected by other bacterial pathogens. Enrollment took place at a single institution in Northeastern Thailand. Adults with culture‐confirmed sepsis (*n* = 29) and uninfected controls (n = 10) were enrolled in the study. Type 2 diabetes is a predisposing factor for Melioidosis, and was represented in equal proportions between the sepsis and control groups. The samples were run on Illumina HT‐12 V3 beadarrays.

GSE26440 Wong *et al*.:^14^ Expression data for derivation of septic shock subgroups. This study aimed to define sepsis subtypes via blood transcriptome profiling and phenotyping. Enrollment took place at 11 different institutions in the USA; children ≤ 10 years old and who presented in the intensive care unit exhibiting paediatric‐specific criteria for septic shock (*n* = 98) and healthy controls (*n* = 32) were enrolled.

### Leucocyte RNAseq reference dataset

A dataset that we previously generated and deposited in GEO was used to assess restriction of the expression of members of the Annexin family across leucocyte populations (Fig. 3). The accession number for this dataset is GSE60424 and the publication of record is from Linsley *et al*.^15^ Specifically, this dataset consisted of RNAseq profiles of neutrophils, monocytes, B‐cells, CD4+ T‐cells, CD8+ T‐cells and natural killer (NK) cells isolated from the blood of healthy controls, patients with type 1 diabetes, amyotrophic lateral sclerosis, sepsis or multiple sclerosis prior to and 24 hr post‐treatment with IFN beta (up to 20 subjects per cell type).

### Statistics

*T*‐test statistics were used throughout for pair‐wise group comparisons and were performed in Microsoft Excel. An F‐test was used to test if the variances of two groups were equal. The level of statistical significance is indicated on the plot: *t*‐test *P*‐values < 0·05*, <0·01**, < 0·001***.

### Literature search strategy

The query used to retrieve the body of literature associated with ANXA3 consisted of a concatenation of its official name, symbol and aliases retrieved from the Entrez Gene, Uniprot and OMIM databases, as follows: ANXA3 [tw] OR ‘Annexin A3’ [tw] OR ‘Inositol 1,2‐Cyclic Phosphate 2‐Phosphohydrolase’ [tw] OR ‘Placental Anticoagulant Protein III’ [tw] OR ‘35‐Alpha Calcimedin’ [tw] OR ‘Lipocortin III’ [tw] OR ‘Annexin‐3’ [tw] OR ‘Annexin 3’ [tw] OR ‘PAP‐III’ [tw] OR ANX3 [tw] OR ‘Annexin III’ [tw] OR ‘Placental Anticoagulant Protein III’ [tw] OR ‘Calcimedin 35‐Alpha’ [tw] AND (annexin [tw] OR phosphohydrolase [tw] OR placental [tw] OR calcimedin [tw] OR ANXA3 [tw] OR Lipocortin [tw] OR anticoagulant [tw] OR calcium [tw]).

The [tw] argument was used to restrict the search to ‘text words’ (title or abstract). The last argument in this query, following the boolean ‘AND’, was designed to minimize the number of false positives that arise with the use of the PAP‐III alias, which stands for Placental Anticoagulant Protein III. Without including these terms, >50 articles were returned that instead referred to PAP smear testing or Pancreatitis Associated Protein III.

## Results

### ANXA3 transcript abundance is robustly increased in septic patients

**Figure 1:**
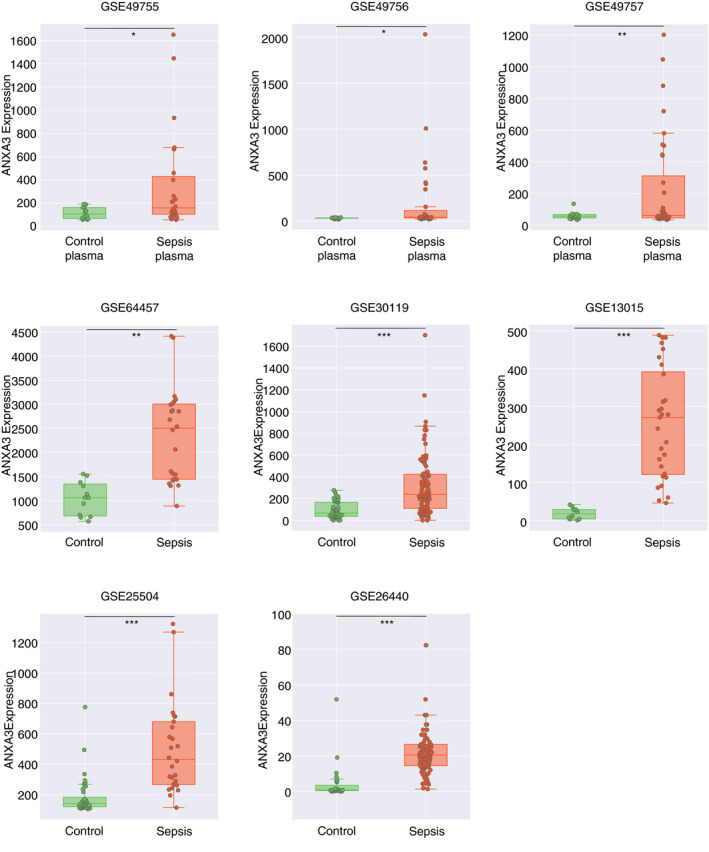
Annexin A3 (ANXA3) is upregulated in septic individuals and in cultured cells exposed to serum/plasma isolated from septic patients. The plots represent ANXA3 transcript abundance, as measured by microarray across different publicly available datasets (see text and Table 1). The three plots along the top correspond to independent replications of an experiment in which neutrophils in culture were exposed to plasma from uninfected control subjects or septic patients for 6 hr (deposited in GEO by Khaenam *et al*. under the GEO ID GSE49755/56/57). The GSE64457 data show the ANXA3 transcript abundance in neutrophils isolated from patients diagnosed with septic shock and neutrophils from healthy controls. The data presented in all other plots derived from studies that profiled the whole blood of septic patients and uninfected controls (all involved paediatric populations except for GSE13015). The level of statistical significance is indicated on each plot: *t*‐test *P*‐values < 0·05*, < 0·01**, < 0·001***.

Once ANXA3 was selected, we ascertained the robustness of the initial observation by examining changes in ANXA3 expression across seven additional public transcriptome datasets (Fig. 1; Table 1; detailed description in the Materials and methods). Out of the seven datasets, two were independent repeats of the neutrophil serum exposure profiling experiment that served as a basis for our initial screen (GSE49756, GSE49757).^2^ The five additional datasets originated from other studies and were contributed to the GEO by Banchereau *et al*. (GSE30119),^10^ Demaret *et al*. (GSE64457),^10^ Dickinson *et al*. (GSE25504),^12^, ^16^ Pankla *et al*. (GSE13015)^13^ and Wong *et al*. (GSE26440).^14^ Of these seven datasets: (i) four were from studies conducted in adults and three in paediatric subjects; (ii) three were from studies that took place in Asia, two in North America, two in Europe; (iii) five were generated using Illumina Beadarrays and two used Affymetrix Genechips; (iv) four used whole blood as starting material and three used purified neutrophils. We observed significant increases in ANXA3 transcript abundance across all the datasets (Fig. 1). This finding suggests that ANXA3 transcript abundance increases in the context of sepsis, regardless of the study and experimental settings.

**Table 1:** Characteristics of the public sepsis datasets used for validation

GSE ID	GSE49755	GSE49756	GSE49757	GSE30119	GSE64457	GSE25504	GSE13015	GSE26440
Title	A Transcriptomic Reporter Assay Employing Neutrophils to Measure Immunogenic Activity of Septic Patients Plasma ‐ Neutrophils, experiment I	A Transcriptomic Reporter Assay Employing Neutrophils to Measure Immunogenic Activity of Septic Patients Plasma ‐ Neutrophils, experiment II	A Transcriptomic Reporter Assay Employing Neutrophils to Measure Immunogenic Activity of Septic Patients Plasma ‐ Neutrophils, experiment III ‐	Genome‐wide analysis of whole blood transcriptional response to community‐acquired *Staphylococcus aureus* infection *in vivo*‐	Marked alterations of neutrophil functions during sepsis‐induced immunosuppression‐	Whole blood mRNA expression profiling of host molecular networks in neonatal sepsis	Genomic Transcriptional Profiling Identifies a Blood Biomarker Signature for the Diagnosis of Septicemic Melioidosis‐	Expression data for derivation of septic shock subgroups
References (PMID)	Khaenam *et al*. (24612859)	Khaenam *et al*. (24612859)	Khaenam *et al*. (24612859)	Banchereau *et al* (22496797)	Demaret *et al*. (27568821)	Dickinson *et al*. (26484146), Smith *et al*. (25120092)	Pankla *et al*. (19903332)	Wong *et al*. (19624809)
Samples	40	49	56	143	23	63	39	130
Species	Homo sapiens	Homo sapiens	Homo sapiens	Homo sapiens	Homo sapiens	Homo sapiens	Homo sapiens	Homo sapiens
Population age demographics	Adult	Adult	Adult	Paediatric	Adult	Paediatric	Adult	Paediatric
Population geographic location	Asia	Asia	Asia	North America	Europe	Europe and Africa	Asia	North America
Entry criteria	Cultured‐confirmed sepsis	Culture‐confirmed sepsis	Culture‐confirmed sepsis	Culture‐confirmed sepsis	Sepsis shock diagnosis	Suspicion of infection, later confirmed by positive cultures	Culture‐confirmed sepsis	Sepsis shock diagnosis
Assay type	Microarray	Microarray	Microarray	Microarray	Microarray	Microarray	Microarray	Microarray
Microarray manufacturer	Illumina	Illumina	Illumina	Illumina	Affymetrix	Illumina	Illumina	Affymetrix
Study type	*In vitro* (cultured with plasma for 6 hr)	*In vitro* (cultured with plasma for 6 hr)	*In vitro* (cultured with plasma for 6 hr)	*In vivo*	*Ex vivo*	*In vivo*	*Ex vivo*	*In vivo*
Cell Type	Neutrophils	Neutrophils	Neutrophils	Whole Blood	Neutrophils	Whole Blood	Whole Blood	Whole Blood
Group A	Uninfected	Uninfected	Uninfected	Control	Healthy Control	Neonatal Sepsis Control	Healthy	Normal Control
Group B	Septic	Septic	Septic	*Staphylococcus aureus* infection	Septic Patient	Infected	Melioidosis	Septic Shock Patient
ANXA3 Exp A	117	34·01	59·09	94·95	1157·40	185·87	18·77	4·17
ANXA3 Exp B	358	223·01	233·59	314·58	2789·04	506·61	253·04	24·91
B/A	3·1	6·55	3·96	3·29	2·41	2·72	11·92	2·19
*t*‐test	< 0·05	0·02	0·002	< 0·001	0·002	< 0·001	< 0·001	< 0·001
*F*‐test	< 0·001	< 0·001	< 0·001	< 0·001	0·48	< 0·001	< 0·001	< 0·001
Dataset GXB Link	http://sepsis.gxbsidra.org/dm3/geneBrowser/show/4000072	http://sepsis.gxbsidra.org/dm3/geneBrowser/show/4000073	http://sepsis.gxbsidra.org/dm3/geneBrowser/show/4000074	http://sepsis.gxbsidra.org/dm3/geneBrowser/show/4000136	http://sepsis.gxbsidra.org/dm3/geneBrowser/show/4000127	http://sepsis.gxbsidra.org/dm3/geneBrowser/show/4000104	http://sepsis.gxbsidra.org/dm3/geneBrowser/show/4000130	http://sepsis.gxbsidra.org/dm3/geneBrowser/show/4000102
ANXA3 GXB Link	http://sepsis.gxbsidra.org/dm3/miniURL/view/Pk	http://sepsis.gxbsidra.org/dm3/miniURL/view/OP	http://sepsis.gxbsidra.org/dm3/miniURL/view/OU	http://sepsis.gxbsidra.org/dm3/miniURL/view/OL	http://sepsis.gxbsidra.org/dm3/miniURL/view/Pj	http://sepsis.gxbsidra.org/dm3/miniURL/view/ON	http://sepsis.gxbsidra.org/dm3/miniURL/view/OT	http://sepsis.gxbsidra.org/dm3/miniURL/view/OS

The fact that we observed an increase in ANXA3 transcript abundance in purified neutrophils is important: changes measured in whole blood could otherwise also be attributed to an increase in relative abundance of this cell population, in the absence of an increase in gene expression. It is also notable that increases in ANXA3 abundance were observed across virtually all age groups, including newborns (GSE25504), as well as among patients with different disease presentations or severities (e.g. in patients where the presentation ranges from skin abscesses, to pneumonia or osteoarticular infection: GSE30119; in patients diagnosed with septic shock: GSE26440, and at both early and late stages of sepsis: GSE64457).

**Figure 2:**
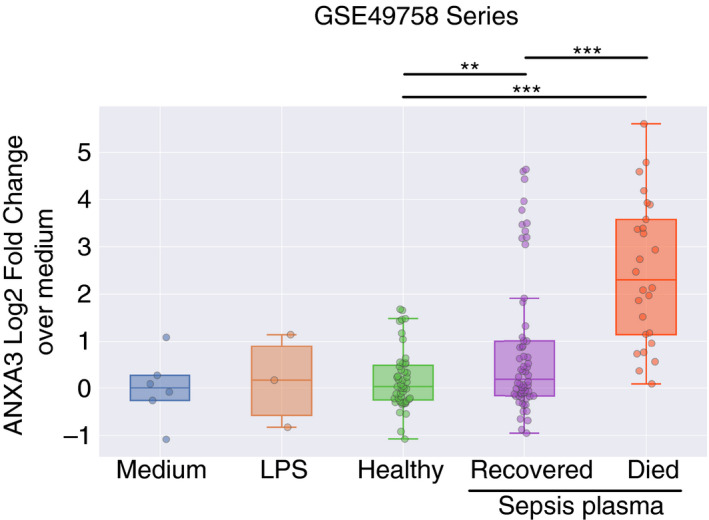
Annexin A3 (ANXA3) upregulation in neutrophils in response to septic plasma correlates with clinical outcomes. The three independent datasets contributed by Khaenam *et al*. (GSE49755/56/57) were combined after normalizing each to the average of its respective controls (neutrophil cultures with medium only). Other conditions included cells exposed to LPS or to plasma from: (i) uninfected controls; (ii) septic patients who responded to treatment and eventually recovered; and (iii) septic patients who did not improve and ultimately succumbed to sepsis. The level of statistical significance is indicated on the plot: *t*‐test *P*‐values < 0·01**, < 0·001***.

By combining the three neutrophil plasma exposure datasets (GSE49755, GSE49756, GSE49757), we could examine a possible association between ANXA3 induction in neutrophils and clinical outcomes (Fig. 2). We found that the abundance of ANXA3 transcripts was increased at much higher levels in response to plasma from patients who eventually died from the infection than in response to plasma from those who recovered. This finding is consistent with the conclusions made by Khaenam *et al*.,^2^ who identified differences in the transcriptome responses between patients with severe and non‐severe sepsis, although an association with mortality was not reported.

Overall, the synthesis of available public data points to a robust increase in ANXA3 abundance in the context of sepsis. This finding is based on neutrophils isolated from septic patients and neutrophils from healthy donors that were exposed to septic plasma *in vivo*. ANXA3 upregulation in sepsis is also observed in the whole blood of patients presenting with different degrees of disease severity and across all age groups, from infancy to adulthood.

### Additional reference datasets inform on ANXA3 restriction and regulation in leucocytes

Next, we accessed gene‐centric expression profiles for additional datasets that are available online through our Gene Expression Browser web application (GXB). Various curated dataset collections are available via this web interface, including one that encompasses 93 transcriptome datasets that are relevant to sepsis (http://sepsis.gxbsidra.org/dm3/geneBrowser/list). Two datasets provided useful perspectives regarding the restriction of ANXA3 expression among circulating leucocytes and its regulation *in vitro* by various immune stimulators.

Annexin A3 is particularly abundant in neutrophils, accounting for ~1% of all cytosolic proteins,^4^ but it has not been described in other immune cell types. We used the available transcriptome datasets to determine the ANXA3 expression profile in leucocyte populations (see Materials and methods for details). We also accessed the transcript abundance profiles of other Annexin family members (accessible via: http://sepsis.gxbsidra.org/dm3/miniURL/view/Or).^9^

**Figure 3:**
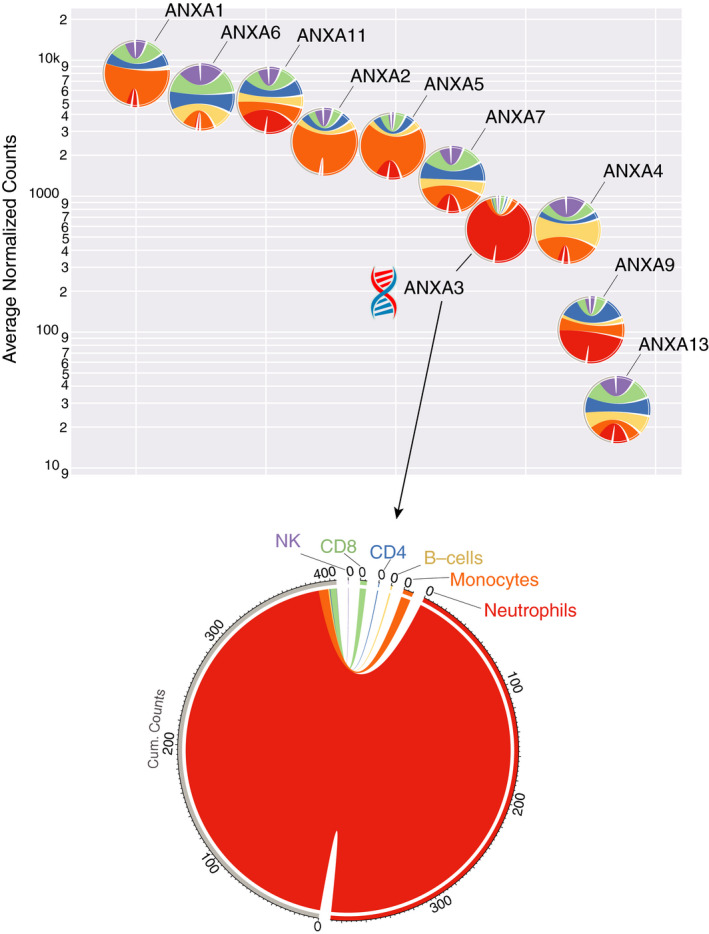
Annexin A3 (ANXA3) shows the most restricted expression to neutrophils of all Annexin family member transcripts. Each chord diagram (circle) presents the relative transcript abundance for a given gene across six leucocyte populations: neutrophils, monocytes, B‐cells, CD4 T‐cells, CD8 T‐cells and NK cells. The colours assigned to each cell population are shown on the ANXA3 diagram (bottom). The predominance of a given colour in a diagram indicates a tendency of expression of the Annexin family member to be preferentially restricted to a given leucocyte population. ANXA3 is predominantly coloured red, indicating the restricted expression of this transcript to neutrophils. Conversely, ANXA2 and ANXA5 are predominantly coloured orange, indicating the preferentially restricted expression of these transcripts to monocytes. Placement of the diagrams along the *y*‐axis indicates the average abundance levels (expressed as normalized counts) of each Annexin family member. Placement along the *x*‐axis reflects the arrangement in descending order of average intensity. The segments on the right are partitioned by cell type, and their size is determined according to the average counts of each population across the 20 study subjects [14 in the case of natural killer (NK) cells]. The ribbons for each segment on the right connect to the same segment on the left, which consists of the sum of average counts (cum. counts). The plots were generated using the ‘Circlize’ R package.^58^ Levels of expression of ANXA3 in neutrophils were found to be significantly higher than in all other cell types (*P* < 0·001; interactive box plot: http://sepsis.gxbsidra.org/dm3/miniURL/view/Pv).

The overall abundance of ANXA3 transcripts across leucocyte populations was lower than for most other Annexin family members: ANXA1, ANXA6 and ANXA11 showed the highest levels of expression (~1 log higher than ANXA3), followed by ANXA2, ANXA5 and ANXA7 (~0·5 log higher; Fig. 3). ANXA3 did, however, exhibit the greatest level of cell specificity among all of the family members, with its expression being almost exclusively restricted to neutrophils (the difference expression levels in neutrophils compared with all other cell types was highly significant, with *P* < 0·001. Data can be visualized and downloaded here: http://sepsis.gxbsidra.org/dm3/miniURL/view/Pv). ANXA9 and ANXA11 were also predominantly restricted to neutrophils. ANXA11, like ANXA3, translocates to the neutrophil granule membrane in a calcium‐dependent manner, and associates with phagosomes.^17^ ANXA2, ANXA5 and ANXA1 were preferentially expressed in monocytes, and their roles in this cell population have been well described in the literature.^18^, ^19^ It is also worth noting that, in line with the results presented earlier, ANXA3 expression was induced in neutrophils and whole blood of the three septic patients included in this dataset (http://sepsis.gxbsidra.org/dm3/miniURL/view/Pl). But it did not appear to be robustly induced in another cell population. Indeed, differences in abundance between leucocyte populations far outweighed any variation observed within a given population and across pathological states.

**Figure 4:**
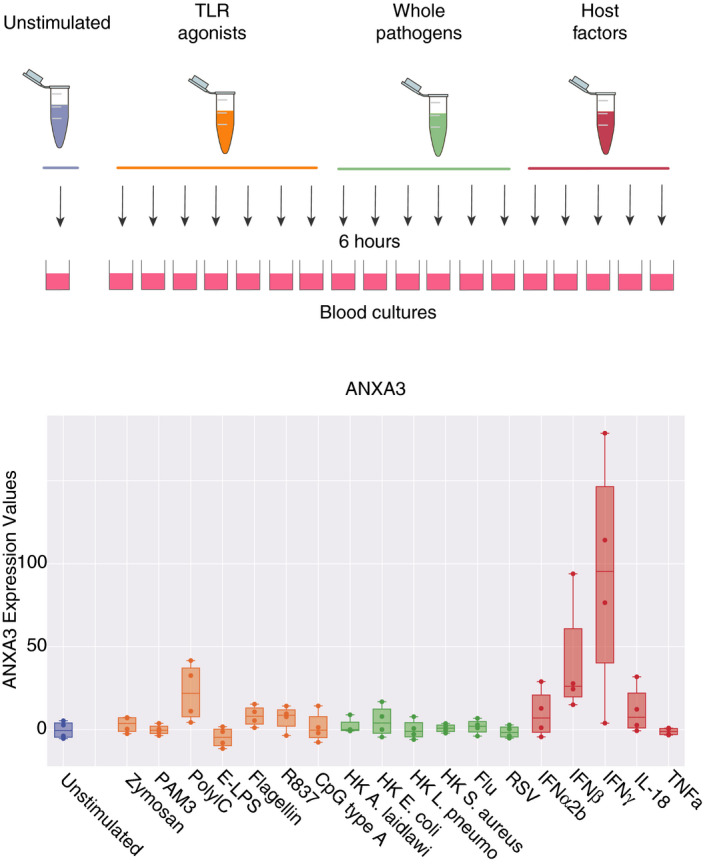
Annexin A3 (ANXA3) transcript abundance *in vitro* changes after exposure to certain immunostimulatory molecules. This reference dataset was contributed to GEO by our group.^59^ Fresh whole blood samples from four healthy subjects were incubated for 6 hr at 37° with one of 18 different stimuli or left unstimulated. The RNAs were then stabilized with Tempus reagent and extracted before processing for microarray analysis. The stimulation conditions included: PAM3, Zymosan, Poly IC, E‐LPS, Flagellin, R837, CpG Type A, heat‐killed *Legionella pneumophila* (HKLP), heat‐killed *Acholeplasma laidlawii* (HKAL), and heat‐killed *Staphylococcus aureus* (HKSA) (all from Invivogen); IL‐18, TNF‐α, IFN‐α2b, IFN‐β, IFN‐γ (all from Peprotech); heat‐killed *Escherichia coli* (in house preparation), live influenza A virus and live respiratory syncytial virus (RSV). Levels of expression under stimulation by interferon (IFN‐α2b, IFN‐β, IFN‐γ) were found to be significantly higher than in all other conditions (*t*‐test, *P* < 0·05).

The availability of a transcriptome dataset from a study in which blood was stimulated *in vitro* with a wide range of immunostimulatory molecules provided an opportunity to evaluate the factors that might drive the increase in ANXA3 expression *in vitro*.^20^ The immunostimulatory molecules included purified pathogen‐derived molecules, host‐inflammatory factors and several species of heat‐killed bacteria that can cause sepsis (Fig. 4).^21^ Surprisingly, ANXA3 was not upregulated in response to the pathogen‐derived or host‐derived pro‐inflammatory molecules. These molecules are potent neutrophil activators. And, indeed, the transcript abundance of some prototypical inflammation markers (such as CCL2 or IL1B) dramatically increased under the same conditions (GXB links: Refs [22, 23]). However, ANXA3 transcript abundance was upregulated in blood cultures following stimulation with interferon (IFN) gamma and, to a lesser degree, IFNβ and poly‐IC (Fig. 4; ANXA3 abundance levels were significantly elevated in IFN‐stimulated conditions compared with all other stimuli, with *t*‐test *P* < 0·05; http://sepsis.gxbsidra.org/dm3/miniURL/view/Pa). This finding is unexpected, as ANXA3 expression has not been reported as being induced by IFN. However, we also found that an increase in ANXA3 transcript abundance was reported in seven out of eight datasets when querying the ‘interferome’ database.^24^ The fold change values ranged from 2·3 to 8·7 (average, 3·9 fold). In the one discordant dataset, the ANXA3 transcript abundance decreased by 14‐fold, but this experiment compared the response of THP1 cells with IFN with and without LPS pretreatment.^25^ No changes were observed in response to IFN in murine cells.

### Interpretation and inferences regarding the potential role of ANXA3 in sepsis

Annexin A3 transcript abundance seems to be robustly increased during sepsis and after exposing neutrophils to septic plasma *in vitro*. Surprisingly, we found that ANXA3 is not induced by canonical host‐derived or pathogen‐derived immune stimulators or by *in vitro* exposure to whole pathogens. We also observed that ANXA3 expression among circulating leucocytes is almost entirely restricted to neutrophils, a property that is unique among the members of the ANXA protein family.

An initial literature search returned no known association between ANXA3 and sepsis. This was confirmed when a more thorough PubMed search was conducted, including all ANXA3 gene names and aliases (see Materials and methods for details). The ANXA3 literature retrieved by this search consisted of 262 entries (as of March 2020). Among those entries, none contained the keyword ‘septic’ or ‘sepsis’ in their titles or abstracts, 20 contained the keywords *‘*neutrophil’ or *‘*neutrophils’, 32 contained the keywords ‘inflammation’ or ‘inflammatory’, and 69 contained the keyword ‘blood’. This simple profiling of the literature confirmed the lack of overlap between the sepsis and ANXA3 literatures.

Notably, earlier transcriptome re‐analyses that included some of the datasets listed above did report ANXA3 transcript abundance as being increased in sepsis and other inflammatory diseases such as systemic arthritis and systemic lupus erythematosus.^26^, ^27^, ^28^ However, these studies, as those upon which they are based, have not attempted to address the biological significance or clinical relevance of changes in ANXA3 abundance during sepsis. Instead, ANXA3 was one among tens of other genes for which changes in abundance were measured and reported. Change in ANXA3 abundance as a result of sepsis was thus not mentioned in the title or abstracts of these studies, but those could be identified via a full text search in Google scholar. While reporting such changes does constitute a form of pre‐existing knowledge, this also illustrates the existence of ‘interpretation gap’ inherent to analyses carried out at more global scales.

We thus endeavoured next to start to address such an interpretation gap on one hand from changes in abundance observed in ancillary reference transcriptome datasets, and on the other hand by inferring from its known functions and involvement in other biological/pathological processes.

Regarding the associations observed with the keywords linked to sepsis (i.e. inflammation, immunity, blood): several recent publications have reported an increased abundance of ANXA3 transcripts in the blood of patients with colorectal cancer, pancreatic cancer,^29^, ^30^ Huntington’s disease, cluster headaches, psoriasis and pneumococcal meningitis.^31^, ^32^, ^33^, ^34^ In the latter study, the context of which is most relevant to the topic of this review, ANXA3 was 1 of 11 markers found to be differentially expressed in the blood of children with meningitis compared with uninfected controls (the initial discovery was made in 12 subjects, and then validated using a targeted polymerase chain reaction panel in a set of 229 subjects).^31^ Relatively few studies have examined a functional role for ANXA3 in immunity and inflammation. In the early 1990s, various research groups investigated the role of ANXA3 in promoting membrane–membrane contact in neutrophils and, more specifically, the fusion of phagocytic vesicle membranes with the membranes of neutrophil granules.^35^, ^36^ Imaging data also showed that ANXA3 redistributed to phagosomes, a phenomenon that was observed in both neutrophils and immature dendritic cells.^35^, ^37^ Given the propensity of ANXA3 to promote granule aggregation in a calcium‐dependent manner, a role in neutrophil degranulation has been suggested,^4^ but not been formally demonstrated. On the basis of these findings, we infer a possible role for ANXA3 in mediating neutrophil microbicidal function and pathogen clearance during sepsis.

Other parts of the ANXA3 literature may also yield additional clues concerning a role for ANXA3 in sepsis. For example, ANXA3 function in tumorigenesis has been an important research focus in recent years. The findings obtained thus far point towards a potential use of ANXA3 as a diagnostic, prognostic and predictive biomarker for cancer patient management.^38^, ^39^, ^40^, ^41^ More relevant to the sepsis profiling data presented and discussed here, mechanistic studies point to a role played by ANXA3 in promoting cancer growth and metastasis.^42^, ^43^, ^44^, ^45^ Notably, a recent study also reported benefits of anti‐ANXA3 monoclonal antibodies in a preclinical mouse model of HCC.^45^ Although ANXA3 expression in many cancer cells seems to correlate directly with the rate of cellular proliferation,^47^, ^48^ the underlying mechanism(s) are undefined. We think that it might be worth investigating a possible role for ANXA3 in extending neutrophil longevity — a phenomenon that has been observed during sepsis and is implicated in end‐organ damage.^49^ It is notable that, on one hand, improved tumour‐cell survival and proliferation was shown to be mediated, in part, by ANXA3‐mediated caspase 3 (CASP3) suppression.^45^, ^50^, ^51^ And in vascular smooth muscle cells a decrease in CASP3 levels was also shown to accompany inhibition of apoptosis and increased proliferation induced by ANXA3.^52^ On the other hand, CASP3 is also well‐known to mediate neutrophil programmed cell death: indeed over 1000 records being returned by the PubMed query: neutrophil* AND (‘Caspase 3’ OR CASP3).^53^, ^54^, ^55^, ^56^, ^57^ Specifically, implications in the regulation of immunopathological processes, including sepsis, have been well documented, with, for instance, reports showing that in activated neutrophils apoptosis can be prevented via CASP3 inhibition.^53^, ^55^ Taken together, this literature might provide sufficient ground to explore a possible role of ANXA3 in the regulation of neutrophil survival, which may have a direct implication on survival in the context of sepsis.

## Conclusions

To date, a role for ANXA3 in sepsis has not been reported in the literature. Our analysis of public transcriptome data supports that ANXA3 transcript abundance is significantly increased in the blood in patients with sepsis; our hypothesis now remains to be elucidated via downstream studies and/or experimentation. Inferences can nevertheless be drawn regarding some of the questions that have been left open.

1. The role played by ANXA3 in neutrophils during sepsis might be beneficial to the host. Indeed, ANXA3 promotes phagolysosome fusion and thus contributes to neutrophil antimicrobial activity.^5^ This function was not investigated in the context of sepsis, but it can be inferred that an increase in ANXA3 abundance in this context may contribute to pathogen clearance and infection resolution.
2. ANXA3 might also have a detrimental effect on the host, by promoting neutrophil survival. This hypothesis is based on the most recently described role of ANXA3 as a factor mediating the proliferation and survival of tumour cells. And, indeed, during sepsis an increase in the neutrophil lifespan may be one of the factors promoting end‐organ damage.^49^
3. The factors responsible for upregulating ANXA3 expression *in vivo* and *in vitro* after exposure to septic plasma remain to be determined. Unexpectedly, *in vitro* blood stimulation data indicate that ANXA3 expression does not increase upon neutrophil activation by pathogen‐derived or host‐derived inflammatory factors. ANXA3 expression in whole blood also increases upon stimulation with IFN or poly:IC. Whether ANXA3 upregulation is mediated by IFN in the context of sepsis is unknown.

Seminal studies exploring the roles of ANXA3 in neutrophil immune biology were published in the 1990s, but were not pursued beyond that decade. We consider that our observations provide sufficient grounds for revisiting and further characterizing the role of ANXA3 in neutrophils, and especially in the context of sepsis. Specifically, the role that ANXA3 may play in enhancing pathogen clearance and prolonging survival of neutrophils could be beneficial during the early phase of the immune response to the infection. At a later stage when the immune response becomes dysregulated, the same properties could however lead to severe tissue damage, organ failure and death.

## Disclosure

The authors declare that they have no conflict of interest.

## Author contributions

Conceptualization: MT, DR, DC, MG. Data curation and validation: MT, JR, MA, BAK, MS, APL, DKB, SM, DR, DC, MG. Visualization: MT, DR, DC, MG. Analysis and interpretation: MT, JR, MA, BAK, MS, APL, DKB, SM, DR, DC, MG. Writing of the first draft: MT, DC, MG. Funding acquisition: DC. Methodology development: MT, MG, DR, DC. Software development and database maintenance: MT. Writing – review & editing: MT, JR, MA, BAK, MS, APL, DKB, SM, DB, WH, SAK, AT, DR, DC, MG. The contributor’s roles listed above follow the Contributor Roles Taxonomy (CRediT) managed by The Consortia Advancing Standards in Research Administration Information (CASRAI) (https://casrai.org/credit/).

## Data Availability

The data that support the findings of this study were derived from the NCBI Gene Expression Omnibus at https://www.ncbi.nlm.nih.gov/geo/, dataset reference numbers are provided throughout the text where appropriate.
